# A Rare Case of p190 BCR-ABL B-cell Acute Lymphocytic Leukemia With Excellent Response to Hyper-CVAD and Ponatinib

**DOI:** 10.7759/cureus.32921

**Published:** 2022-12-25

**Authors:** Nathan DeRon, Sereena Jivraj, Hannah McCreight, Austin Permann, Babaniji Oluwadamilola

**Affiliations:** 1 Internal Medicine, Methodist Health System, Dallas, USA; 2 Internal Medicine, Texas Christian University Brunett School of Medicine, Fort Worth, USA; 3 Internal Medicine, Texas College of Osteopathic Medicine, Fort Worth, USA

**Keywords:** rash, case report, philadelphia chromosome, leukemia diagnosis, all, acute lymphoblastic leukemia, b-all

## Abstract

Acute lymphoblastic leukemia (ALL) is a hematologic cancer that begins in the bone marrow and results in an overproduction of lymphocytes. It can present as palpable purpura, which is also seen in many other pathologies, including vasculitides such as IgA vasculitis. We present a case of a 52-year-old male who presented to our hospital from an outside facility specifically for plasma exchange for treating a previously diagnosed IgA vasculitis. After being admitted for further evaluation, it was noted that the patient had a diffuse petechial non-blanching purpuric rash bilaterally covering the lower extremities, trunk, upper extremities, and tongue. The patient was also noted to have severe pancytopenia. Fluorescence in situ hybridization (FISH) demonstrated the presence of t(9:22), indicating Philadelphia chromosome rearrangement. The patient was diagnosed with ALL. The patient underwent induction chemotherapy and was continued on hyper-CVAD protocol with intrathecal chemotherapy. The patient appeared to respond well to treatment and is currently undergoing subsequent intermittent chemotherapy. In this case, the diagnosis of B-cell ALL (B-ALL) blast crisis was pivotal in providing the correct therapy to this patient, and the case demonstrated that even rare presentations of B-ALL in this population with rare mutations responds avidly to tyrosine kinase inhibitors.

## Introduction

B-cell acute lymphoblastic leukemia (B-ALL) is a disease entity involving a heterogeneous group of genetically variable lymphoid neoplasms. These genetic variations cause the uninhibited production of cells from B-lymphoid progenitors [1]. It is important to recognize that, according to the World Health Organization, acute lymphoblastic leukemia (ALL) and lymphoblastic lymphoma are disease entities with overlapping clinical presentations, and diagnosis or classification typically does not distinguish between the two [2]. ALL represents only 12% of all leukemia cases worldwide [3]. It is a disease primarily found in children. More than 75% of cases occur in children less than six years old [3]. However, there is a second, modest peak in individuals greater than or equal to 60 years old [4]. In younger patients, B-ALL has a higher survival rate. Several factors play important roles in the development of B-ALL, including ionizing radiation, medications and chemicals, and genetic abnormalities [5,6].

There is a vast array of clinical manifestations that may take form due to B-ALL. Many of these signs and symptoms are due to direct invasion of the medullary bone marrow. This may include anemia, leukopenia, and thrombocytopenia leading to clinical signs such as easy bruising and severe fatigue [5-7]. Symptoms such as headache, vision changes, neck stiffness, confusion, and facial numbness can occur with central nervous system invasion [8-10].

## Case presentation

The patient is a 52-year-old Caucasian male with a history of supraventricular tachycardia status post ablation and dual-chamber implantable cardioverter defibrillator placement, type 2 diabetes mellitus, hypertension, chronic kidney disease, and a presumed diagnosis of IgA vasculitis without any confirmatory biopsy or pathology information. The patient reported a three-year history of bilateral lower extremity episodic rash progressing to the trunk and bilateral arms but sparing the palms and soles. The patient reported attending outpatient clinic visits during these episodes and was treated with corticosteroids with successful remission of rash symptoms. However, there was no history of severe pancytopenia during these clinic visits. Prior to presentation to our facility, the patient reported two to three weeks of a similar rash associated with abdominal pain, nausea, and vomiting. The patient also reported severe malaise, fatigue, and generalized weakness during this time, such that he was unable to ambulate around his house as usual due to excessive weakness. Upon further questioning, the patient reported an approximately 40-pound unintentional weight loss over the previous four weeks as well as one episode of fever and subjective chills. The patient denied further symptoms such as hearing changes, vision changes, cough, gross hematuria, and lower extremity edema.

Due to his previous presumed diagnosis of IgA vasculitis, the patient was again diagnosed with this disorder and started on corticosteroid therapy at the outside facility. The patient was also provided a total of three units of packed red blood cells due to severe anemia. The patient continued to deteriorate at the outside facility and was transferred to our facility for a higher level of care for the presumed need for plasma exchange in the setting of severe IgA vasculitis. Upon arrival, the patient was found to have the lab values noted in Table 1.

**Table 1:** Complete blood count, comprehensive metabolic panel, hemolysis, and inflammatory lab results demonstrating leukocytosis with severe bicytopenia RDW - red blood cell distribution width

	Parameter	Values at outside facility	Values upon arrival
Complete blood count	White blood cells	18.7 k/uL	5.4 k/uL
	Hemoglobin	3.7 gm/dL	6.2 gm/dL
	Hematocrit	11.20%	18.10%
	Mean corpuscular volume	89.9 fL	89.2 fL
	RDW	16.80%	15.50%
	Platelets	4 k/uL	1 k/uL
	Relative segmental neutrophils	5%	-
	Absolute segmental neutrophils	0.9 k/uL	-
	Relative lymphocytes	95%	-
	Absolute lymphocytes	17.8 k/uL	-
	Relative monocytes	0%	-
	Relative eosinophils	0%	-
	Relative basophils	0%	-
Comprehensive metabolic panel	Glucose	411 mg/dL	403 mg/dL
	Blood urea nitrogen	35 mg/dL	46 mg/dL
	Creatinine	2.4 mg/dL	2.3 mg/dL
	Sodium	133 mmol/L	134 mmol/L
	Potassium	6.4 mmol/L	4.6 mmol/L
	Chloride	100 mmol/L	102 mmol/L
	Carbon dioxide	18.0 mmol/L	20 mmol/L
	Calcium	8.5 mg/dL	8.5 mg/dL
	Corrected calcium	-	9.1 mg/dL
	Albumin	3.4 gm/dL	3.2 gm/dL
	Aspartate transaminase	66 IU/L	49 IU/L
	Alanine transaminase	41 IU/L	26 IU/L
	Alkaline phosphatase	92 IU/L	64 IU/L
	Total bilirubin	0.7 mg/dL	-
Hemolysis and inflammatory lab results	Haptoglobin	294 mg/dL	-
	Lactate	1481 IU/L	-
	Erythrocyte sedimentation rate	26 mm/hr	-
	C-reactive protein	5.3 mg/dL	-

Flow cytometry was performed on the complete blood count gathered at our facility. A striking 48% of cells were found to be blast positive for the CD10 cell marker, as illustrated in Table 2.

**Table 2:** Flow cytometry results demonstrating cluster of differentiation positivity suggestive of B-cell malignancy

Marker	Result
CD10	POSITIVE
CD45	DIM POSITIVE
CD34	POSITIVE
CD117	NEGATIVE
MPO	NEGATIVE
TDT	POSITIVE
CD79A	POSITIVE
CD19	POSITIVE
CD22	POSITIVE
CD123	POSITIVE
HLA-DR	POSITIVE
CD61	NEGATIVE
CD13	NEGATIVE
CD33	NEGATIVE
CD11B	NEGATIVE
FMC7	DIM POSITIVE
CD38	POSITIVE

Further evaluation revealed 61.56% BCR-ABL p190 transcript with fluorescence in situ hybridization (FISH) reporting t(9;22) and gain of RUNX1 (21q) as demonstrated in Table 3.

**Table 3:** Fluorescence in situ hybridization results revealing two positive gene rearrangements confirming B-ALL due to a chromosome 9:22 translocation B-ALL - B-cell acute lymphoblastic leukemia

Chromosomal abnormality	Result
BCR-ABL1, t(9:22)	58% rearrangement
Gain of RUNX1 (21q)	50%-55% rearrangement
PML/RARA	Negative
CBFB	Negative
KMT2A (MLL)	Negative
RUNX1T1/RUNX1	Negative
RUNX1 (21q22.12)	Negative

The patient was diagnosed with B-ALL. The patient was determined to be a poor transplant candidate due to multiple comorbidities. Chemotherapy with cyclophosphamide, vincristine, doxorubicin (Adriamycin), and dexamethasone was initiated in a hypofractionated approach known as hyper-CVAD. Combination therapy with dasatinib was also initiated. Induction therapy was complicated by diabetic ketoacidosis and metabolic acidemia treated with insulin infusion and sodium bicarbonate. The patient was also treated for both hypocalcemia and hyperphosphatemia during the initial hospital course. Renal function improved, but the patient subsequently developed fevers attributed to neutropenia. Subsequent blood cultures grew Gamella species, and intravenous cefepime was initiated with an adequate response. Repeat blood cultures did not show any further bacterial growth. The patient required multiple transfusions of both packed red blood cells and platelets during the hospital course. Intrathecal chemotherapy with methotrexate was also administered without complication. The patient was subsequently discharged with instructions to present in one month for the next round of therapy.

The patient received monthly hyper-CVAD treatments with ponatinib, rituximab, and intrathecal methotrexate or cytarabine for four additional sessions with major molecular response and low positive BCR-ABL quantification after the second subsequent cycle of therapy. The patient was considered in complete remission after the fourth subsequent cycle.

## Discussion

Establishing the diagnosis of B-cell ALL quickly and efficiently is imperative to provide patients with timely and adequate treatment. The hallmark of diagnosis for B-ALL is characteristic morphology in cells from sources including peripheral blood, bone marrow, or lymph node tissue. The cell morphology is variable but generally includes either dense nuclear chromatin or dispersed chromatin with multiple nucleoli in the absence of Auer rods.

In BCR-ABL t(9;22) translocation, the subsequent fusion proteins directly depend on the breakpoint in the BCR gene and may result in variants including major p210, minor p190, and micro p230 proteins [11, 12]. The p190 variant demonstrated in this case is quite rare in chronic leukemia/lymphoma [13, 14], and therefore, therapy targeted at this mutation is continuing to undergo investigation. Acute leukemia does have a somewhat higher prevalence of p190 mutation.

As with other forms of BCR-ABL positive ALL, tyrosine kinase inhibitors are a stalwart of therapy. Options include both imatinib and dasatinib, among others. The disease-free survival rate with tyrosine kinase therapy is typically at least 70% regardless of combination with intensive chemotherapy [15]. Complications from tyrosine kinase or chemotherapy of ALL include thrombosis, tumor lysis syndrome, bleeding, and infection. However, side effects can also be specific to each tyrosine kinase inhibitor. Therefore, it is typically recommended that patients receive therapy with close supervision by the administering provider.

A final important note regarding this case is the illustration of the importance of fully investigating the differential diagnosis in similar cases. The patient in this case was initially transferred to a higher level of care for consideration of plasma exchange, but the true diagnosis was B-ALL rather than the presumed IgA vasculitis. If further investigation by flow cytometry and pathology review of peripheral blood smear were not pursued, a significant delay in the initiation of therapy may have resulted in the worsening of the patient's condition or untimely death.

## Conclusions

Due to the low incidence of p190 BCR-ABL transformation to B-ALL, the diagnosis is often obscured and may be delayed due to confounding findings. Any delay in initiation of therapy for this disease may represent further harm to the patient, given that therapy for positive BCR-ABL mutations is very effective and minimally harmful relative to other types of immunotherapy and chemotherapy. This case demonstrates the importance of fully exploring the differential diagnosis and obtaining as much medical history as possible for the goal of caring for the patient. It is important that clinicians recognize the variable clinical presentations of B-ALL to improve the alacrity of therapy initiation for the eventual remission of this treatable disease.

## References

[1] Onciu M (2009). Acute lymphoblastic leukemia. Hematol Oncol Clin North Am.

[2] Arber DA, Orazi A, Hasserjian R (2016). The 2016 revision to the World Health Organization classification of myeloid neoplasms and acute leukemia. Blood.

[3] Redaelli A, Laskin BL, Stephens JM, Botteman MF, Pashos CL (2005). A systematic literature review of the clinical and epidemiological burden of acute lymphoblastic leukaemia (ALL). Eur J Cancer Care (Engl).

[4] Dores GM, Devesa SS, Curtis RE, Linet MS, Morton LM (2012). Acute leukemia incidence and patient survival among children and adults in the United States, 2001-2007. Blood.

[5] Hunger SP, Mullighan CG (2015). Acute lymphoblastic leukemia in children. N Engl J Med.

[6] Pui CH, Yang JJ, Hunger SP (2015). Childhood acute lymphoblastic leukemia: progress through collaboration. J Clin Oncol.

[7] Inaba H, Greaves M, Mullighan CG (2013). Acute lymphoblastic leukaemia. Lancet.

[8] Bleyer WA (1988). Central nervous system leukemia. Pediatr Clin North Am.

[9] Ingram LC, Fairclough DL, Furman WL, Sandlund JT, Kun LE, Rivera GK, Pui CH (1991). Cranial nerve palsy in childhood acute lymphoblastic leukemia and non-Hodgkin's lymphoma. Cancer.

[10] Kraigher-Krainer E, Lackner H, Sovinz P, Schwinger W, Benesch M, Urban C (2008). Numb chin syndrome as initial manifestation in a child with acute lymphoblastic leukemia. Pediatr Blood Cancer.

[11] Dai X, Tian F, Xu Z, Kong X, Jiang P, Xia W, Zhu X (2021). Philadelphia chromosome-positive acute lymphoblastic leukemia: a case report. Ann Palliat Med.

[12] Gatti A, Movilia A, Roncoroni L, Citro A, Marinoni S, Brando B (2018). Chronic myeloid leukemia with P190 BCR-ABL translocation and persistent moderate monocytosis: a case report. J Hematol.

[13] Pardanani A, Tefferi A, Litzow MR, Zent C, Hogan WJ, McClure RF, Viswanatha D (2009). Chronic myeloid leukemia with p190BCR-ABL: prevalence, morphology, tyrosine kinase inhibitor response, and kinase domain mutation analysis. Blood.

[14] Gandhe N, Vekaria M, Dabak V (2021). A rare case of p190 BCR-ABL chronic myeloid leukemia with a very good response to tyrosine kinase inhibitors. Cureus.

[15] Schultz KR, Carroll A, Heerema NA (2014). Long-term follow-up of imatinib in pediatric Philadelphia chromosome-positive acute lymphoblastic leukemia: Children's Oncology Group study AALL0031. Leukemia.

